# Long-Term Efficacy Outcomes of Natalizumab vs. Fingolimod in Patients With Highly Active Relapsing-Remitting Multiple Sclerosis: Real-World Data From a Multiple Sclerosis Reference Center

**DOI:** 10.3389/fneur.2021.699844

**Published:** 2021-08-23

**Authors:** Marina Boziki, Christos Bakirtzis, Virginia Giantzi, Styliani-Aggeliki Sintila, Stylianos Kallivoulos, Theodora Afrantou, Ioannis Nikolaidis, Panagiotis Ioannidis, Theodoros Karapanayiotides, Ioanna Koutroulou, Dimitrios Parissis, Nikolaos Grigoriadis

**Affiliations:** 2^*nd*^ Neurological University Department, American Hellenic Educational Progressive Association (AHEPA) General Hospital, Aristotle University of Thessaloniki (A.U.TH.), Thessaloniki, Greece

**Keywords:** relapsing-remitting multiple sclerosis, natalizumab, fingolimod, disease-modifying treatment, annualized relapse rate, highly-active multiple sclerosis

## Abstract

**Background:** Natalizumab (NTZ) and fingolimod (FTY) are second-line disease modifying treatments (DMTs) approved for Relapsing – Remitting Multiple Sclerosis (RRMS). Few studies are available on a direct comparison between NTZ and FTY, based on post-marketing experience, with conflicting results and reporting relatively short follow-up period.

**Aim:** We hereby report real-world experience of a MS Center with respect to NTZ vs. FTY comparison in terms of efficacy and safety, referencing long-term follow-up.

**Methods:** We used retrospective data for all patients that received 2nd-line treatment NTZ (since May 2007) or FTY (since September 2011). Primary endpoints were, among others, annual EDSS score (mean change from baseline), time to disability worsening or improvement, Annualized Relapse Rate (ARR) after 12 and 24 months and upon total treatment duration, time to first relapse and time to radiological progression.

**Results:** A total of 138 unmatched patients, 84 treated with NTZ and 54 treated with FTY were included. Following Propensity Score (PS) matching, 31 patients in each group were retained. Mean follow-up period for NTZ- and FTY-treated patients was 4.43 ± 0.29 and 3.59 ± 0.32 years (*p* = 0.057), respectively. In the matched analysis, time to disability improvement and time to disability worsening was comparable between groups. A higher proportion of patients remained free of relapse under NTZ, compared to FTY (Log Rank test *p* = 0.021, HR: 0.25, 95% CI: 0.08–0.8), as well as free of MRI activity (Log Rank test *p* = 0.006, HR: 0.26, 95% CI: 0.08–0.6). Treatment discontinuation due to MRI activity was significantly higher for FTY-treated patients compared to NTZ (Log Rank test *p* = 0.019, HR: 0.12, 95% CI: 0.05–0.76).

**Conclusion:** Our results indicate toward NTZ superiority with respect to relapse and MRI activity outcomes. The fact that NTZ-treated patients may achieve long-standing clinical and radiological remission points toward the need for long follow-up data.

## Introduction

Natalizumab (NTZ) and fingolimod (FTY) are second-line disease-modifying treatments (DMTs), European Medicines Agency (EMA) approved for Relapsing–Remitting Multiple Sclerosis (RRMS) (1, 2), a classification based on the safety profile of these agents. Both treatments were shown to be effective in controlling clinical and MRI activity in patients with RRMS with highly active disease at diagnosis. Although there is no consensus on the definition for highly active RRMS (3), NTZ and FTY are indicated in patients with RRMS for whom at least one DMT has previously proven ineffective and/or exhibit rapidly evolving severe RRMS defined by two or more disabling relapses in 1 year, one or more Gd(+) lesions on brain MRI, or a significant increase in T2 lesion load as compared to a previous recent MRI (1, 2). The use of NTZ has significantly been affected by the occurrence of Progressive multifocal leukoencephalopathy (PML), a rare but severe adverse event linked to anti-JCV (John Cunningham virus) Ab (antibody) seropositivity, prior use of immunosuppressants, and prolonged (>2 years) exposure to NTZ (4). PML risk stratification has further been implemented in clinical practice according to EMA guidelines and based on the anti-JCV Ab index as well as the duration of exposure to NTZ (5). In this respect, NTZ administration is subjected to weighted risk-benefit estimation for the patient, in the clinical practice. The use of FTY is being affected by the risk of opportunistic infections linked to lymphopenia, macular edema, rare cardiologic abnormalities, and adverse events stemming from the drug's mode of action (6).

More recently, several newly-available treatments for Relapsing Multiple Sclerosis (RMS) and highly-active RRMS have been approved by the EMA (7–9). These treatments are either monoclonal antibodies (alemtuzumab, ocrelizumab) targeting immune cell populations via complement- and/or antibody-dependent cytotoxicity (CDC/ADCC) (10, 11), or, as in the case of cladribine, a purine analog that interferes with cell proliferation (12). Alemtuzumab, an anti-CD52 monoclonal antibody, effectively depletes T- and B-cells from the peripheral blood (10), whereas ocrelizumab, an anti-CD20 monoclonal antibody, selectively targets B-cell populations and a small fraction of anti-CD20-bearing T-cells (11). These treatments, although exhibiting a differential depletion profile with respect to the cell populations affected and the duration of their biological effect, are collectively considered as newer highly effective treatments and have drastically contributed a new approach in the management of MS. The principle of pulsed immune reconstitution in the context of early aggressive treatment for MS has been advocated as an attractive alternative to classic escalation treatment schemes and has been linked with long-term disease remission in carefully selected patients (13, 14). However, potential adverse events of these treatments, resulting mainly from the prolonged immune reconstitution kinetics, limit their use and underline the necessity of personalized treatment decisions (15, 16). The use of the traditionally regarded as second-line DMTs, namely, NTZ and FTY, remains central in the management of highly-active RRMS, as dictated by the long-term experience of the medical community with these agents and the overall favorable safety profile, compared to the newly available highly effective agents.

Available studies on a direct comparison between NTZ and FTY, are based on post-marketing experience, with partly conflicting results (17–27), and few meta-analyses (28, 29). More specifically, the majority of the existing literature indicates natalizumab superiority with respect to markers of clinical and radiological activity (17–20, 22). In two studies, natalizumab superiority was not retained following propensity score (PS) matching and correction of the analysis taking into consideration confounding factors stemming from baseline characteristics of the two cohorts, respectively (21, 23). In one study, the effect of NTZ and FTY on disease clinical outcomes was comparable (27). In this respect, treatment choice in clinical practice is mostly empirical, with anti-JCV Ab seropositivity status and route of administration remaining the main determining factors. Moreover, the results of existing studies include a relatively short follow-up period of ~2 years. We hereby report real-world experience of a multiple sclerosis (MS) Center with respect to NTZ vs. FTY comparison in terms of efficacy and safety, referencing long-term follow-up.

## Materials and Methods

### Inclusion/Exclusion Criteria

All patients included in the present study were followed by the Multiple Sclerosis Center of the 2nd Department of Neurology of the Aristotle University of Thessaloniki in AHEPA University General Hospital. We used retrospective data for all patients that received second-line treatment NTZ (since May 2007) or FTY (since September 2011) and who either discontinued treatment or were currently under treatment (as for August 2020). All patients started NTZ or FTY due to failure of first-line agents [interferons (IFNs) and/or glatiramer acetate] or at treatment-naïve state due to highly active MS at diagnosis, according to EMA label. All patients upon NTZ or FTY treatment initiation were older than 18 years. Treatment with immunosuppressants in the previous year and progressive MS were exclusion criteria. A minimum NTZ or FTY treatment duration of 12 months was necessary for inclusion. Moreover, patients with lost-to-follow-up status during NTZ/FTY treatment were not included. NTZ/FTY treatment initiation was retrospectively regarded as the baseline.

### Data Collection

All demographic, clinical, and MRI data were recorded in paper and electronically in the MS database of the Center (iMED until May 2020 and MDS since June 2020). An Expanded Disability Status Scale (EDSS) score was reported at baseline and every 3 months for all patients included in the study as well as clinical evaluation regarding the type of the disease with respect to possible progression onset. As a relapse, a new or worsening neurologic symptom with at least 24-h duration confirmed by neurological examination following the exclusion of fever and/or infection was considered. A relapse occurring within 3 months of NTZ or FTY onset was not taken into account for annualized relapse rate (ARR) estimation. Brain and cervical Magnetic Resonance Imaging (MRI) data, as well as thoracic MRI, where available, were collected before NTZ/FTY initiation and annually thereafter. Brain and cervical MRI data were available for all patients at all time points. MRI studies were conducted in different facilities, as in routine clinical practice, but were all evaluated by the treating Neurologists of the Center, with at least 5-year experience in treating patients with MS. Where electronic files of MRI scans were available, a record was retained in the Center's MRI database. JCV Ab status evaluation was conducted by STRATIFY JCV™ (Unilabs, Copenhagen, Denmark) for patients before second-line treatment initiation, whereas for NTZ treated patients the EMA guide in JCV Ab status monitoring and PML risk stratification was followed. For all patients discontinuing NTZ or FTY, the exact reason for discontinuation was recorded [e.g., PML concern, EDSS increase and/or secondary progressive multiple sclerosis (SPMS) disease course, treatment inefficacy, adverse event, pregnancy planning, and patient's will].

### Patient Consent and Ethical Declaration

The study was conducted in accordance with the Helsinki Declaration. All participants provided written informed consent. The study received the approval of the Bioethics Committee of the School of Medicine of the Aristotle University of Thessaloniki (Approval Nr. 5321/23-2-2021).

### Outcomes

Primary endpoints were as follows:

- Annual EDSS score
- Time to disability worsening, defined as 1 point of EDSS increase (0.5 points if baseline EDSS ≥ 5.5 and 1.5 points if baseline EDSS = 0.0), confirmed after 6 months;
- Time to disability improvement (defined as an EDSS score decrease of ≥1 point, or ≥1.5 points in case baseline EDSS was 0, confirmed after 6 months);
- Annualized relapse rate ARR after 12 and 24 months
- Annualized relapse rate (ARR) during total treatment duration
- Time to first relapse
- Time to treatment discontinuation due to breakthrough disease (clinical activity)
- Nr of new/enlarging T2 lesions with respect to previous brain and cervical scan on annual MRI
- Nr of T1 gadolinium (Gd+) lesions on annual brain and cervical MRI scan
- Time to radiological progression/MRI activity (defined as the presence of ≥1 new/enlargingT2 lesion with respect to previous brain MRI and/or the presence of ≥1 gadolinium Gd+ lesion) annual brain and cervical MRI scan
- Time to treatment discontinuation due to MRI activity.

### Statistical Analysis

For continuous variables, normality was assessed by a Kolmogorov-Smirnoff test prior to the variables' comparison between the two cohorts. We compared continuous variables by the use of non-parametric Mann–Whitney test and dichotomous and/or categorical variables by the use of Chi-square. For the analysis of unmatched cohorts with respect to mean EDSS, ARR, and MRI activity, and in order to minimize potentially significant imbalances at baseline, we investigated mean parameter change vs. baseline by the use of paired samples *T*-tests. Values were presented as mean ± standard error of the mean. Moreover, for the unmatched cohorts with respect to mean EDSS, ARR, and MRI activity, mixed models for repeated measures were used according to which gender, age (years), MS duration (years), ARR in the precedent year, degree of brain MRI activity at baseline (number of new/enlarging T2 and Gd+ lesions), and baseline EDSS scores were used as covariates. Furthermore, in order to compare the two cohorts following minimization of imbalance at baseline, we used propensity score (PS) 1:1 exact matching method, without replacement, with a caliper of 0.1. Covariates used for PS estimation were as follows: gender, age (years), MS duration (years), ARR in the precedent year, degree of brain MRI activity at baseline (number of new/enlarging T2 and Gd+ lesions), and baseline EDSS score. Anti-JCV Ab status was not included in the PS calculation because not all patients starting NTZ since 2007 performed the test. We assessed the degree of imbalance between matched and unmatched cohorts by calculating measurements of effect size estimation, namely, Mean Standardized Difference (MSD/Cohen's *d*) for continuous variables and Cramer's *V* for dichotomous/categorical variables. A logistic regression model with the parameters used for PS estimation as independent variables were used in order to explore potential variables associated with NTZ or FTY treatment before and after PS matching. We compared survival time endpoints using Kaplan–Meier curves (log rank test) for matched and unmatched cohorts. Moreover, we estimated hazard ratios (HRs) and relative 95% CI using proportional hazards model adjusted (a) by all covariates used for PS calculation and (b) by PS for unmatched cohorts and adjusted by all covariates used for PS calculation for matched cohorts. Also, for unmatched cohorts, a Cox Regression analysis was conducted following inverse probability weighting, adjusted by all covariates used for PS calculation. The analysis was conducted by the use of SPSS IBM v. 25. A significance level of 0.05 was taken into account. For the comparison of baseline characteristics, as well as for the comparison of the mean parameter change vs. baseline by the use of serial paired-samples *T*-test for EDSS and MRI parameters, the *p*-value Bonferrroni's correction for multiple comparisons was applied.

## Results

### Study Population

**Figure 1:**
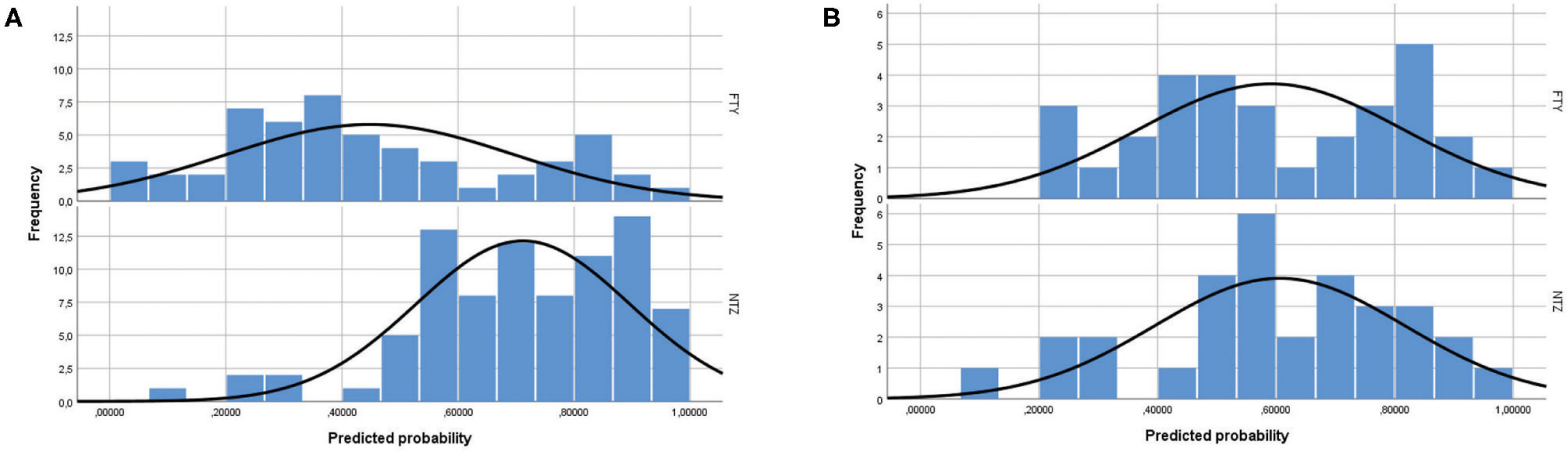
Propensity score distribution in natalizumab and fingolimod patients **(A)** before and **(B)** after propensity score matching. NTZ, natalizumab; FTY, fingolimod.

**Figure 2:**
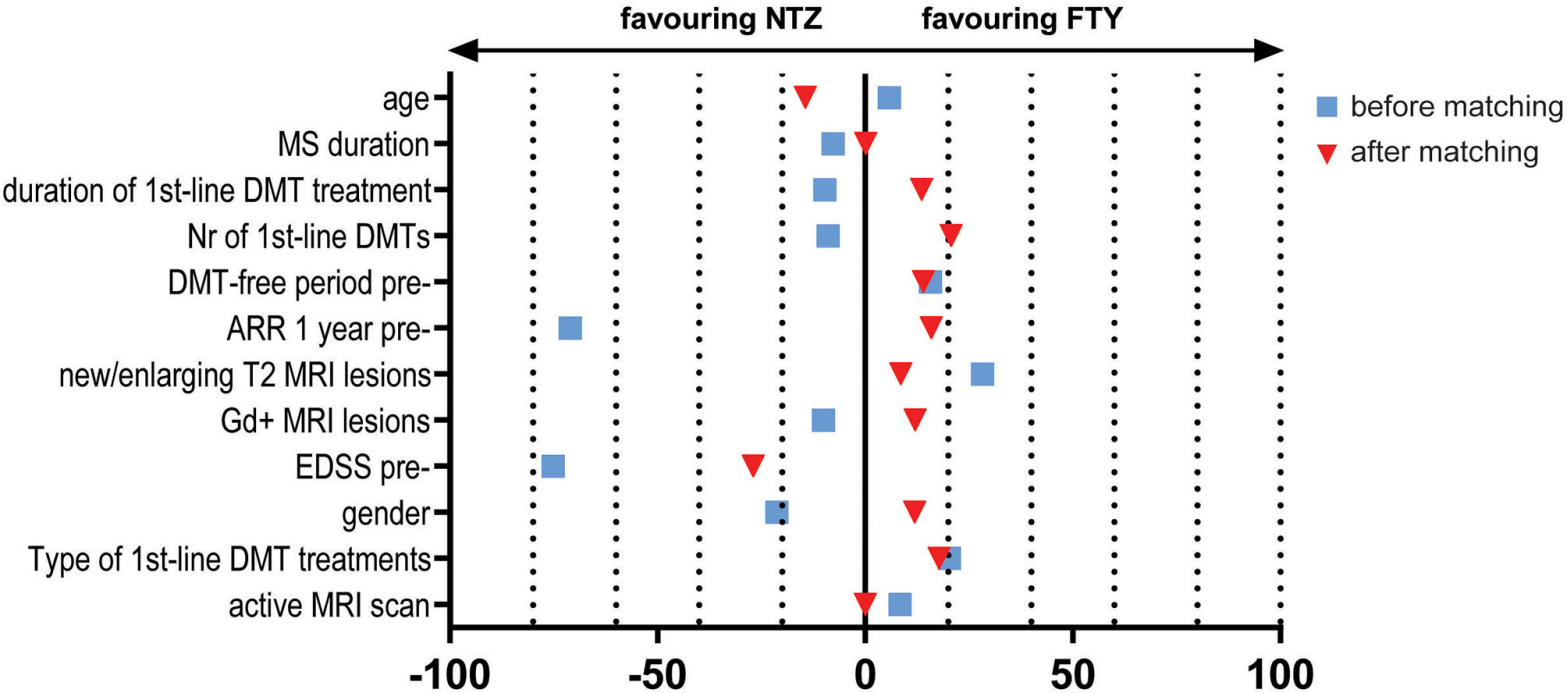
Effect size estimation e.g., Mean Standardized Difference/Cohen's d for continuous variables and Cramer's V for dichotomous/categorical variables, for baseline variables before (blue squares) and after (red triangles) propensity score matching. NTZ, natalizumab; FTY, fingolimod; MRI, Magnetic Resonance Imaging; DMTs, disease-modifying treatments; EDSS, Expanded Disability Status Scale; pre-, before NTZ/FTY; Gd, gadolinium; ARR, annualized relapse rate; Nr, number; MS, Multiple Sclerosis.

The study included a total of 138 unmatched patients: 84 treated with NTZ and 54 treated with FTY. Mean Standardized Difference for PS between the two groups before matching was 1.21 (mean ± SD for NTZ: 0.72 ± 0.18, FTY: 0.45 ± 0.25, *p* < 0.001), and it was reduced to 0.09 following matching (mean ± SD for NTZ: 0.61 ± 0.21 vs. FTY: 0.59 ± 0.22, *p* = 0.783) (Figure 1). Following matching, 31 patients in each group were retained. The reduction in the size of the cohorts after PS matching is primarily attributed to the imbalance of the unmatched cohorts, especially with respect to the ARR in the year before NTZ/FTY onset as well as the EDSS score at baseline (NTZ/FTY onset) (Table 1; Figure 2). Matched cohorts exhibited comparable demographic and clinical characteristics (Table 1). Baseline variables exhibited a degree of imbalance based on standardized differences before matching (for absolute values min: 5.8; max 75.14; range 69.34; mean ± standard error of mean: 23.53 ± 6.96), whereas the degree of imbalance was reduced (<20%) with the exception of the number of first-line DMTs received pre- (20.71) and the EDSS score (26.95), following matching (for absolute values min: 0.1; max 26.95; range 26.85; mean ± standard error of mean: 13.02 ± 2.21) (Table 1; Figure 2). The logistic regression model used for PS estimation indicated that the ARR pre- (OR: 4; 95% C.I. 1.93–8.32, *p* < 0.001) and the EDSS at baseline (OR: 1.96; 95% C.I. 1.39–2.75, *p* < 0.001) were factors associated with NTZ or FTY allocation before PS matching, whereas no factors were associated with NTZ or FTY allocation following PS matching. In the NTZ group, all patients were followed for at least 1 year and 68 patients for 2 years. In the FTY cohort, 54 patients were followed for 1 year and 37 patients for 2 years. Overall, the mean follow-up period for NTZ-treated patients was 4.43 ± 0.29 years, whereas for FTY-treated patients it was 3.59 ± 0.32 years (*p* = 0.057). In the matched groups, the mean follow-up period for NTZ-treated patients was 4.28 ± 0.45 years, whereas, for FTY-treated patients, it was 3.53 ± 0.43 years (*p* = 0.231). A baseline brain MRI scan was performed within 3 months before NTZ/FTY onset. With the exception of one patient in the NTZ group and three patients in the FTY group, overall patients underwent brain MRI scans annually. In total, 17 (20.2%) of NTZ-treated and 34 (63%) of FTY-treated patients were not tested for anti-JCV Ab throughout the treatment duration. Anti-JCV Ab testing was performed in few patients under FTY for reasons of PML risk assessment in the clinical practice, although essentially FTY treatment is linked with minimal PML risk, and no PML risk stratification guideline for FTY-treated patients is available. For NTZ-treated patients, the percentage of patients that were not tested for ant-JCV Ab status is attributed to patients that received NTZ during the early period of the treatment availability (2007–2011). Moreover, due to the same reason, 64 (76.2%) patients that received NTZ were not tested for anti-JCV Ab at baseline. However, the majority of NTZ-treated patients were tested for anti-JCV Ab during the treatment duration.

**Table 1:** Baseline clinical and demographic characteristics of patients under natalizumab and fingolimod before and after propensity score matching.

**Baseline characteristics**	**Before matching**				**After matching**			
	**NTZ (*N* = 84)**	**FTY (*N* = 54)**	***MSD***/*V*	p^*^	**NTZ (*N* = 31)**	**FTY (*N* = 31)**	***MSD***/*V*	p^*^
Gender (male/female)	23/61	12/42	5.8	n.s.	5/26	8/23	11.9	n.s.
Age	36.11 ± 1.07	34.07 ± 1.25	−21.24	n.s.	36.23 ± 1.4	35.06 ± 1.52	−14.38	n.s.
Disease duration (years)	9.76 ± 0.62	9.28 ± 0.95	−7.76	n.s.	10.9 ± 1.05	10.91 ± 1.32	0.15	n.s.
First-line DMT treatment duration (years)	5.01 ± 0.41	4.64 ± 0.54	−9.73	n.s.	5 ± 0.71	5.55 ± 0.73	13.59	n.s.
Number of first-line DMT treatments	1.24 ± 0.06	1.19 ± 0.09	−8.99	n.s.	1.13 ± 0.1	1.26 ± 0.12	20.71	n.s.
Type of first-line DMT treatments (IFNs/GA/both)	52/3/23	25/6/9	20.3	n.s.	18/1/6	14/3/7	17.8	n.s.
DMT-free period pre-(months)	5.74 ± 1.52	8.56 ± 3.12	15.74	n.s.	6.14 ± 2.71	8.87 ± 4.14	14.11	n.s.
ARR 1 year pre-	1.58 ± 0.06	1.15 ± 0.09	−71.01	** <0.001**	1.32 ± 0.1	1.42 ± 0.12	15.95	n.s.
Patients with active MRI Scan, N (%)	49 (58.33)	36 (66.66)	8.4	n.s.	18 (58.06)	21 (67.74)	0.1	n.s.
Number of New/enlarged T2 lesions (brain & cervical MRI)	1 ± 0.24	1.69 ± 0.37	28.23	n.s.	1.06 ± 0.37	1.26 ± 0.44	8.58	n.s.
Number of Gd+ lesions (brain & cervical MRI)	1.93 ± 0.35	1.63 ± 0.35	−10.04	n.s.	1.65 ± 0.51	2 ± 0.55	12.05	n.s.
EDSS score	3.81 ± 0.15	2.73 ± 0.17	−75.14	** <0.001**	3.58 ± 0.24	3.21 ± 0.26	−26.95	n.s.

*NTZ, natalizumab; FTY, fingolimod; MSD/V, Mean Standardized Difference or Cramer's V; DMT, disease-modifying treatment; IFN, interferon; GA, glatiramer acetate; ARR, annualized relapse rate; MRI, magnetic resonance imaging; EDSS, Expanded Disability Status Scale. Numbers represent mean ± standard error of mean*;

p*, following Bonferroni's correction for multiple comparisons; n.s., non-significant. Comparisons with a p value <0.001 are indicated in bold.

### Treatment Withdrawal and Safety

Anti-JCV Ab was detected during the treatment period in 35 of 84 (41.67%) patients treated with NTZ and in 14 of 54 (25.93%) patients treated with FTY. In total, 31 of 84 (36.9%) patients under NTZ and 6 of 54 (11.11%) patients under FTY were negative throughout the treatment duration. For 33 (39.29%) patients under NTZ, anti-JCV Ab seropositivity was the main reason for treatment withdrawal. For two patients under NTZ that tested positive for anti-JCV Ab, treatment discontinuation was not suggested due to low index value. One patient positive for anti-JCV Ab developed PML. Treatment discontinuation had been suggested for this patient. Overall, treatment discontinuation occurred earlier on average for 29 FTY-treated patients compared to 70 patients under NTZ, however, the difference in the mean treatment duration did not reach statistical significance (treatment duration in months: 38.17 ± 4.38 vs. 49.8 ± 3.75, *p* = 0.094). Reasons for treatment discontinuation were mainly PML concern in 29 patients (34.52%), SPMS course and/or EDSS increase in 15 patients (17.86%), patient's will in 12 cases (14.29%), inefficacy in 10 cases (11.9%), pregnancy planning in 2 cases (2.4%) and insurance issues in 1 (1.2%) case for the NTZ-treated group. One patient developed PML (1.2%). For the FTY-treated group, reasons for treatment discontinuation were inefficacy in 13 (24.07%) cases, lymphopenia in 12 (22.22%) cases, SPMS course and/or EDSS increase in 3 (5.56%) patients, and pregnancy planning for 1 (1.85%) case. Two patients (2.38%) in the NTZ-treated group experienced adverse events with respect to infections, namely, recurrent urinary tract infections and herpes zoster, respectively. In the first case, the adverse events were managed via symptomatic treatment and did not consist reason for discontinuation. In the second case herpes zoster was a secondary reason for discontinuation, together with anti-JCV seropositivity status and PML concern. Nine patients (16.67%) in the FTY-treated group experienced adverse events with respect to infections, namely, recurrent urinary tract infections. Lymphopenia of grade that did not require treatment discontinuation was evident in all patients under FTY, with the exception of the 12 patients for whom lymphopenia dictated treatment discontinuation due to safety concerns. Apart from infections and lymphopenia, no other adverse event was present in the FTY-treated cohort. Mean time (in years) of treatment withdrawal due to relapse and/or MRI activity did not differ between NTZ- (2.92 ± 0.51) and FTY- (3.05 ± 0.59, *p* = 0.878) treated patients.

### Unmatched Cohorts

#### Baseline Characteristics

In the unmatched cohort analysis, patients under NTZ exhibited an increased mean EDSS score compared to FTY-treated patients at baseline (NTZ vs. FTY: 3.81 ± 0.15 vs. 2.73 ± 0.17, *p* < 0.001). Moreover, patients under NTZ exhibited a higher mean ARR the year before treatment onset relative to the patients under FTY (NTZ vs. FTY: 1.58 ± 0.06 vs. 1.15 ± 0.09, *p* < 0.001). Patients under FTY exhibited a comparable mean number of new/enlarging T2 lesions on brain MRI at baseline, to NTZ-treated patients (NTZ vs. FTY: 0.68 ± 0.18 vs. 1.22 ± 0.26, *p* = n.s.). Moreover, no difference was observed between NTZ- and FTY-treated patients with respect to the mean number of gadolinium-enhancing lesions at baseline for brain (NTZ vs. FTY: 1.62 ± 0.33 vs. 1.37 ± 0.33, *p* = n.s.) and cervical (NTZ vs. FTY: 0.31 ± 0.1 vs. 0.26 ± 0.08, *p* = n.s.) MRIs. Similarly, no significant difference was observed with respect to new/enlarging T2 lesions between NTZ- and FTY-treated patients at baseline for brain (NTZ vs. FTY: 0.68 ± 0.18 vs. 1.22 ± 0.26, *p* = n.s.) and cervical (NTZ vs. FTY: 0.32 ± 0.11 vs. 0.46 ± 0.15, *p* = n.s) MRI.

#### Disability, ARR, and MRI Activity: Analysis at Point Estimates

**Figure 3:**
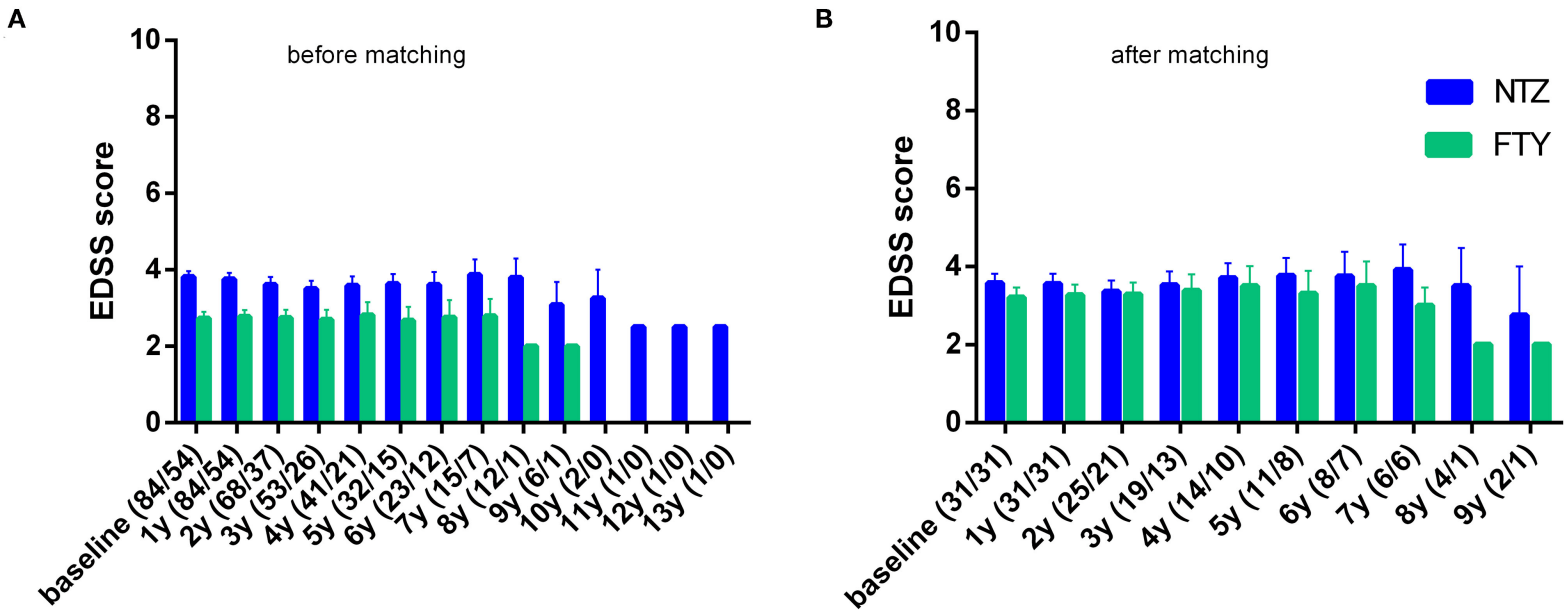
Mean annual expanded disability status scale of natalizumab and fingolimod cohorts at baseline and during the whole follow-up **(A)** before and **(B)** after matching. NTZ, natalizumab; FTY, fingolimod. Analysis was conducted at point estimation by exploring mean change from baseline for each treatment group.

In order to minimize the impact of different baseline cohort activities, the unmatched cohort analysis was conducted by investigating change vs. baseline for each treatment group, with respect to EDSS, ARR, and MRI activity parameters. In the first year of treatment, patients under NTZ and under FTY did not exhibit alterations with respect to mean EDSS score, compared to baseline (for NTZ: 3.81 ± 0.15 vs. 3.76 ± 0.16, *p* = n.s.; for FTY: 2.73 ± 0.17 vs. 2.77 ± 0.18, *p* = n.s). Also, in the second year of treatment patients under NTZ (*N* = 68) and under FTY (*N* = 37) did not exhibit alterations with respect to mean EDSS score compared to baseline (for NTZ: 3.61 ± 0.2 vs. 3.68 ± 0.16, *p* = n.s.; for FTY: 2.74 ± 0.21 vs. 2.68 ± 0.2, *p* = n.s.) (Figure 3A).

**Figure 4:**
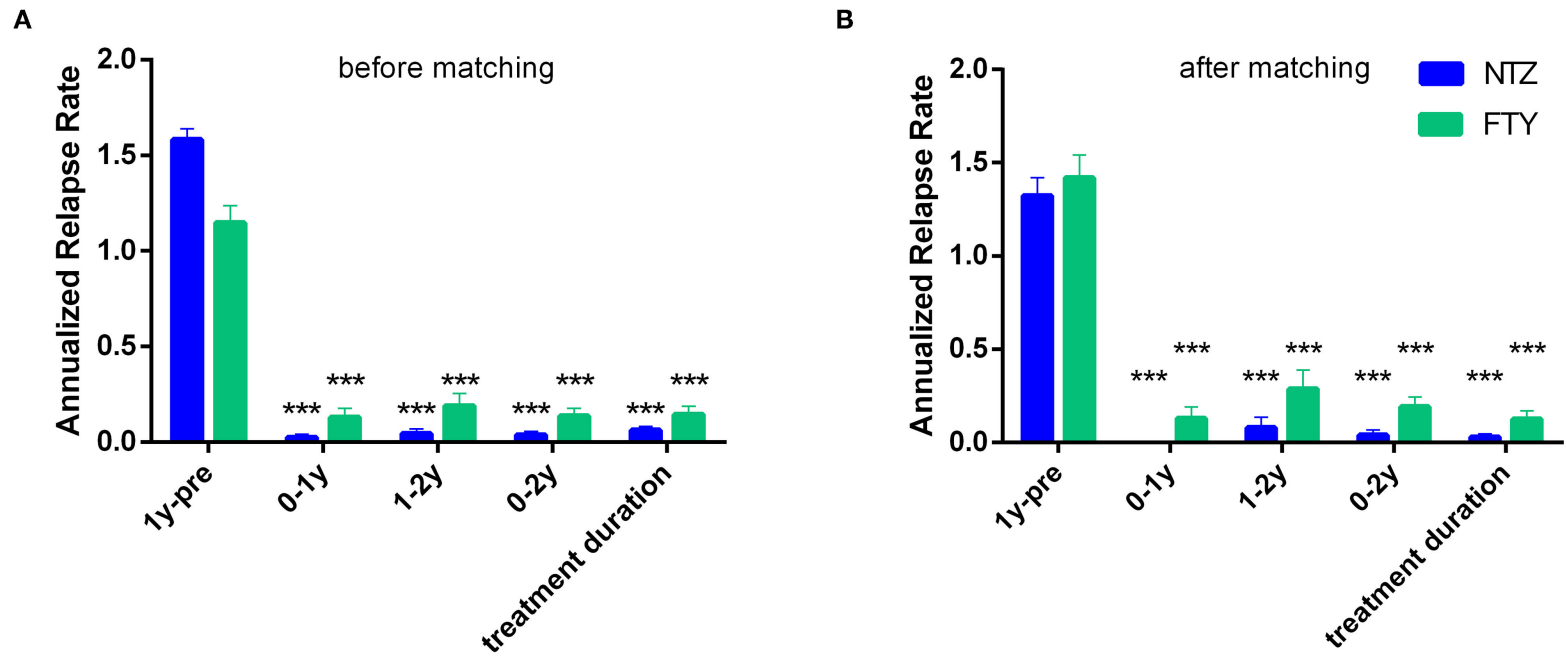
Mean annualized relapse rate of natalizumab and fingolimod cohorts in the year before natalizumab/fingolimod treatment onset (1y-pre; baseline), referencing the first year of treatment (0–1y), referencing the second year of treatment (1–2y), referencing the first and second year of treatment (0–2y), as well as referencing the overall follow-up period under natalizumab/fingolimod treatment (treatment duration). **(A)** before and **(B)** after matching. Statistical significance indicates mean change from baseline (1y pre-). NTZ: natalizumab; FTY: fingolimod; ^***^p < 0.001.

For the NTZ-treated patients, there was a significant mean ARR reduction compared to baseline, referencing year 0–1, year 1–2 (*N* = 68), years 0–2 (*N* = 68), as well as overall NTZ-treatment duration (mean ARR for year 0–1 vs. baseline: 0.02 ± 0.02 vs. 1.58 ± 0.06, *p* < 0.001; for year 1–2 vs. baseline: 0.04 ± 0.03 vs. 1.58 ± 0.06, *p* < 0.001; for years 0–2 vs. baseline: 0.04 ± 0.02 vs. 1.57 ± 0.06, *p* < 0.001 and for overall NTZ treatment duration vs. baseline: 0.06 ± 0.02 vs. 1.58 ± 0.06, *p* < 0.001). Also for the FTY-treated patients, there was a significant mean ARR reduction compared to baseline, referencing year 0–1, year 1–2 (*N* = 37), years 0–2 (*N* = 37), as well as overall FTY-treatment duration (mean ARR for year 0–1 vs. baseline: 0.13 ± 0.05 vs. 1.15 ± 0.09, *p* < 0.001; for year 1–2 vs. baseline: 1.16 ± 0.11 vs. 0.19 ± 0.07, *p* < 0.001; for years 0–2 vs. baseline: 0.14 ± 0.04 vs. 1.16 ± 0.11, *p* < 0.001 and for overall FTY treatment duration vs. baseline: 0.14 ± 0.04 vs. 1.15 ± 0.09, *p* < 0.001) (Figure 4A).

**Figure 5:**
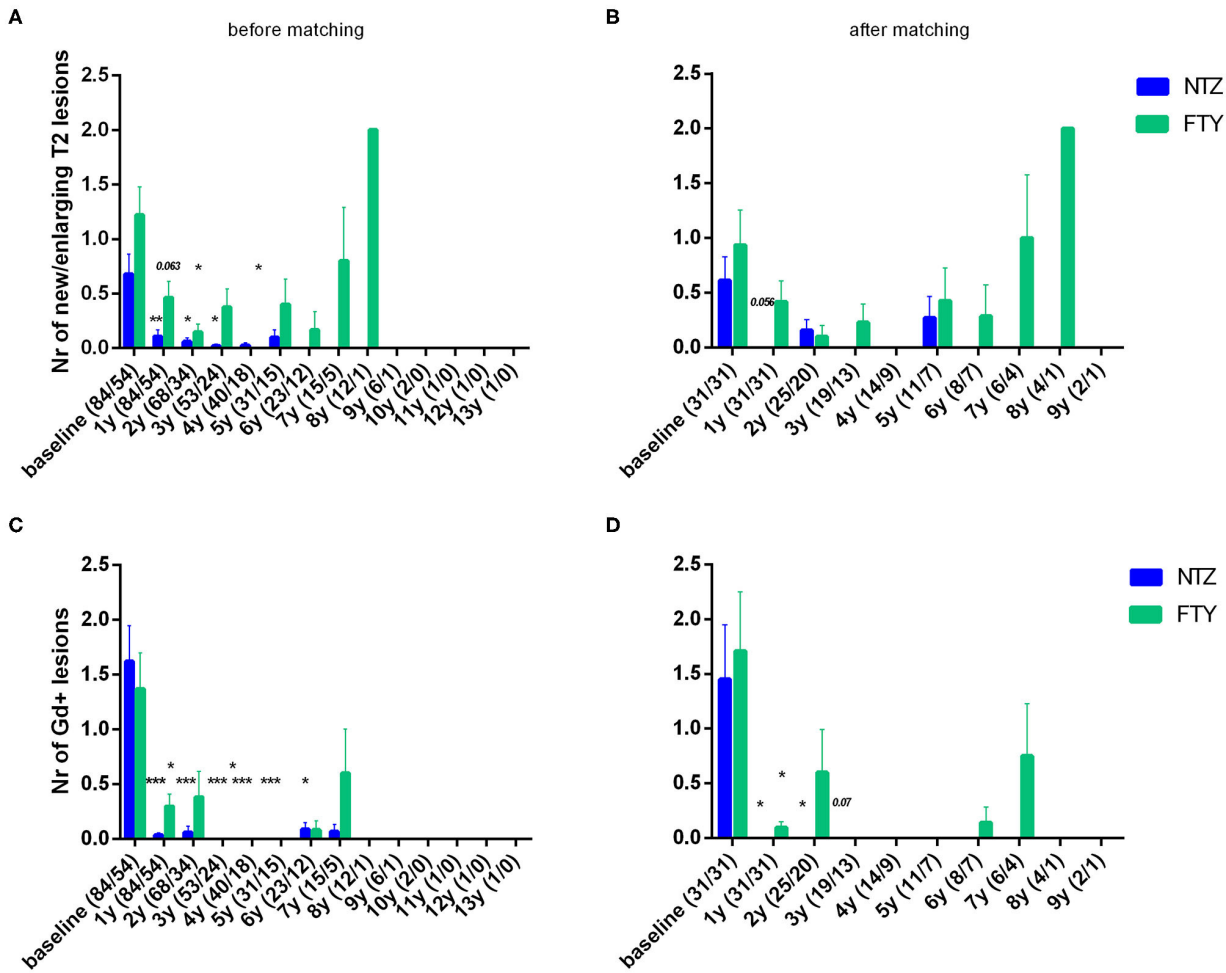
Mean number of new/enlarging T2 lesions **(A,B)** and gadolinium-enhancing lesions **(C,D)** on annual brain MRI of natalizumab and fingolimod cohorts at baseline and during the whole follow-up **(A,C)** before and **(B,D)** after matching. Statistical significance indicates mean change from baseline. NTZ, natalizumab; FTY, fingolimod; Nr, Number; Gd, gadolinium; ^*^p < 0.05;^**^p < 0.005; ^***^p < 0.001.

Patients under NTZ exhibited significant mean reduction from baseline with respect to the number of new/enlarging T2 lesions on brain MRI at annual point estimates from year 1 to year 4 (at year 1 vs. baseline: 0.11 ± 0.06 vs. 0.68 ± 0.18, *p* = 0.007; at year 2 vs. baseline: 0.06 ± 0.04 vs. 0.78 ± 0.22, *p* = 0.014) (Figure 5A). Patients under FTY exhibited a significant mean reduction from baseline with respect to the number of new/enlarging T2 lesions on brain MRI at point estimate year 2 (year 2 vs. baseline: 0.15 ± 0.07 vs. 1.09 ± 0.31, *p* = 0.021) (Figure 5A). A similar effect, overall more significant for NTZ, was observed with respect to the number of gadolinium-enhancing lesions on brain MRIs for NTZ- and FTY- treated patients (Figure 5B) as well as with respect to the number of new/enlarging T2 lesions and the number of gadolinium-enhancing lesions on first-year cervical MRI (Supplementary Figures 1A,B).

#### Disability, Relapse, and MRI Activity: Mixed Models for Repeated Measures

For NTZ-treated patients, with respect to EDSS, a mixed model for repeated measure was overall statistically non-significant (−2 Restricted Log Likelihood = 1,484.016, *p* = n.s.). With respect to ARR, a mixed model for the repeated measure was overall statistically significant (−2 Restricted Log Likelihood = 31.775, *p* < 0.001) with the difference in ARR pre- and post-NTZ treatment initiation being reduced ~1.54 times (*p* < 0.001), whereas it did not differ between the first and the second year of the follow-up. For MRI activity parameters mixed models for the repeated measure were overall statistically non-significant for NTZ-treated patients.

For FTY-treated patients, with respect to EDSS, a mixed model for the repeated measure was overall statistically non-significant (−2 Restricted Log Likelihood = 623.547, *p* = n.s.). With respect to ARR, a mixed model for the repeated measure was overall statistically significant (−2 Restricted Log Likelihood = 184.455, *p* < 0.001) with the difference in ARR pre- and post-FTY treatment initiation being reduced ~0.96 times (*p* < 0.001), whereas it did not differ between the first and the second year of the follow-up. For MRI activity parameters mixed models for the repeated measure were overall statistically non-significant for FTY-treated patients.

#### Disability, Relapse, and MRI Activity–Survival Time Endpoints

**Figure 6:**
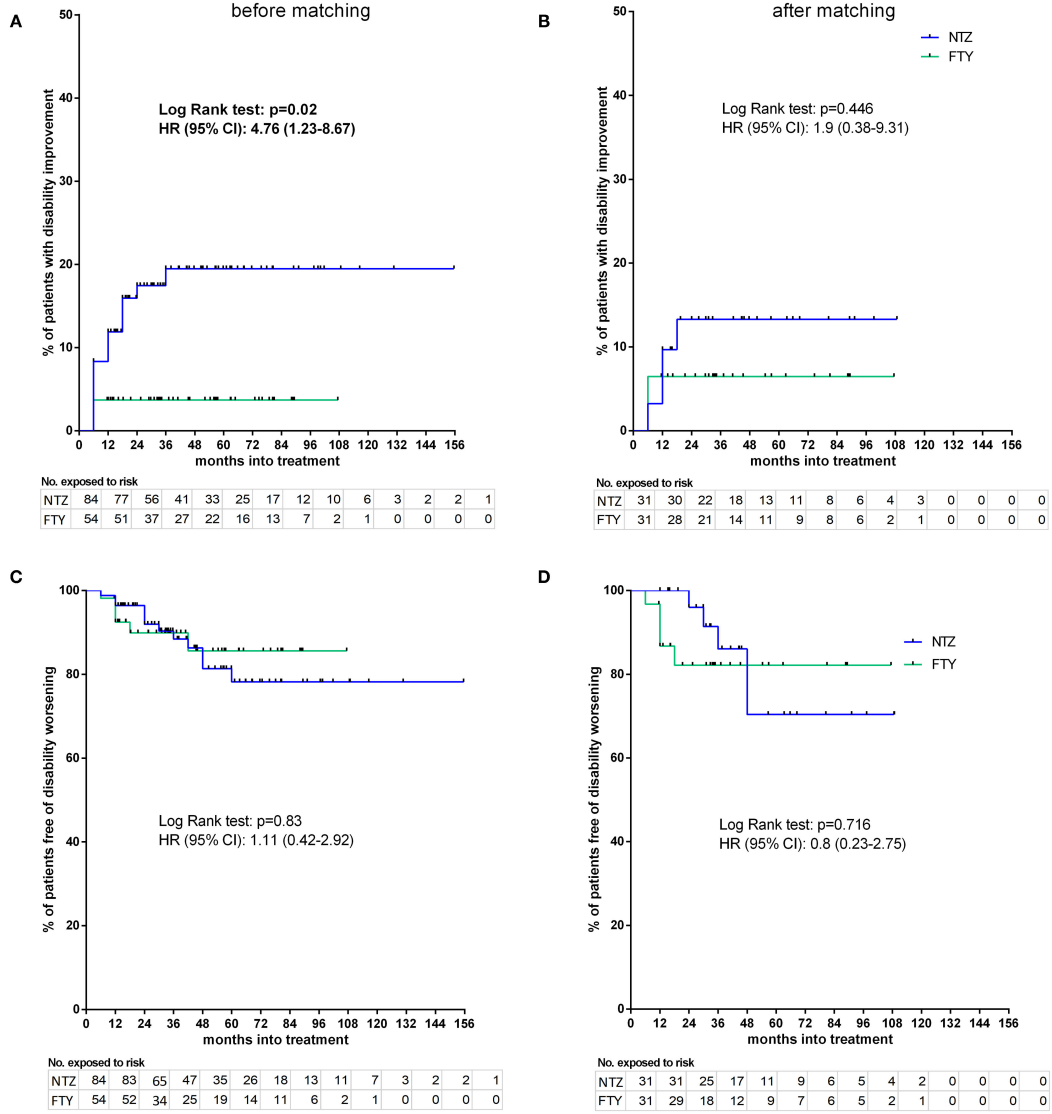
Kaplan–Meier survival estimates for the time to disability improvement **(A,B)** and time to disability worsening **(C,D)** before **(A,C)** and after **(B,D)** matching. HR, hazard ratio; CI, confidence interval; No., number; NTZ, natalizumab; FTY, fingolimod.

**Figure 7:**
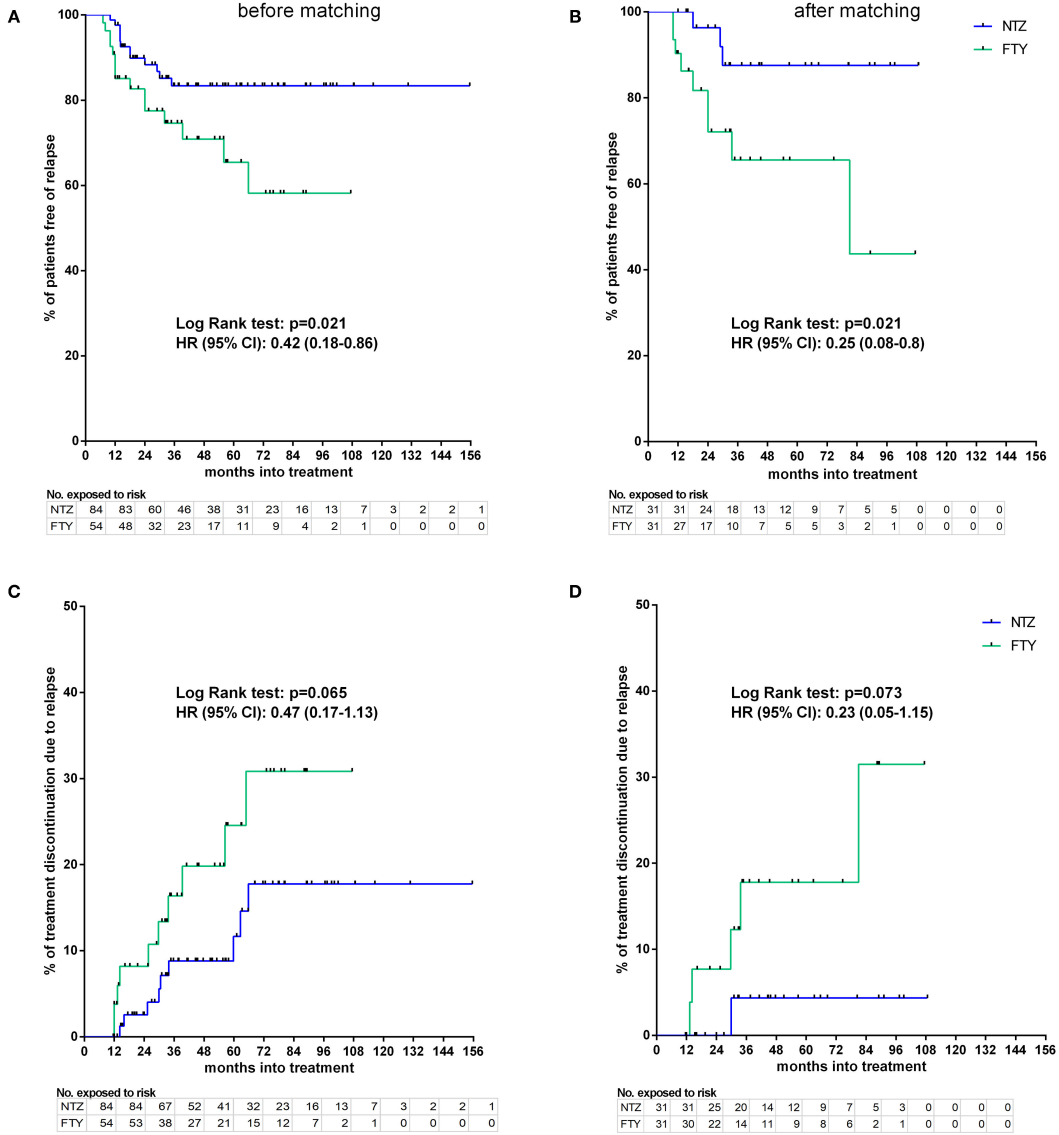
Kaplan–Meier survival estimates for the time to relapse under treatment **(A,B)** and time to treatment discontinuation due to relapse **(C,D)** before **(A,C)** and after **(B,D)** matching. HR, hazard ratio; CI, confidence interval; No., number; NTZ, natalizumab; FTY, fingolimod.

**Figure 8:**
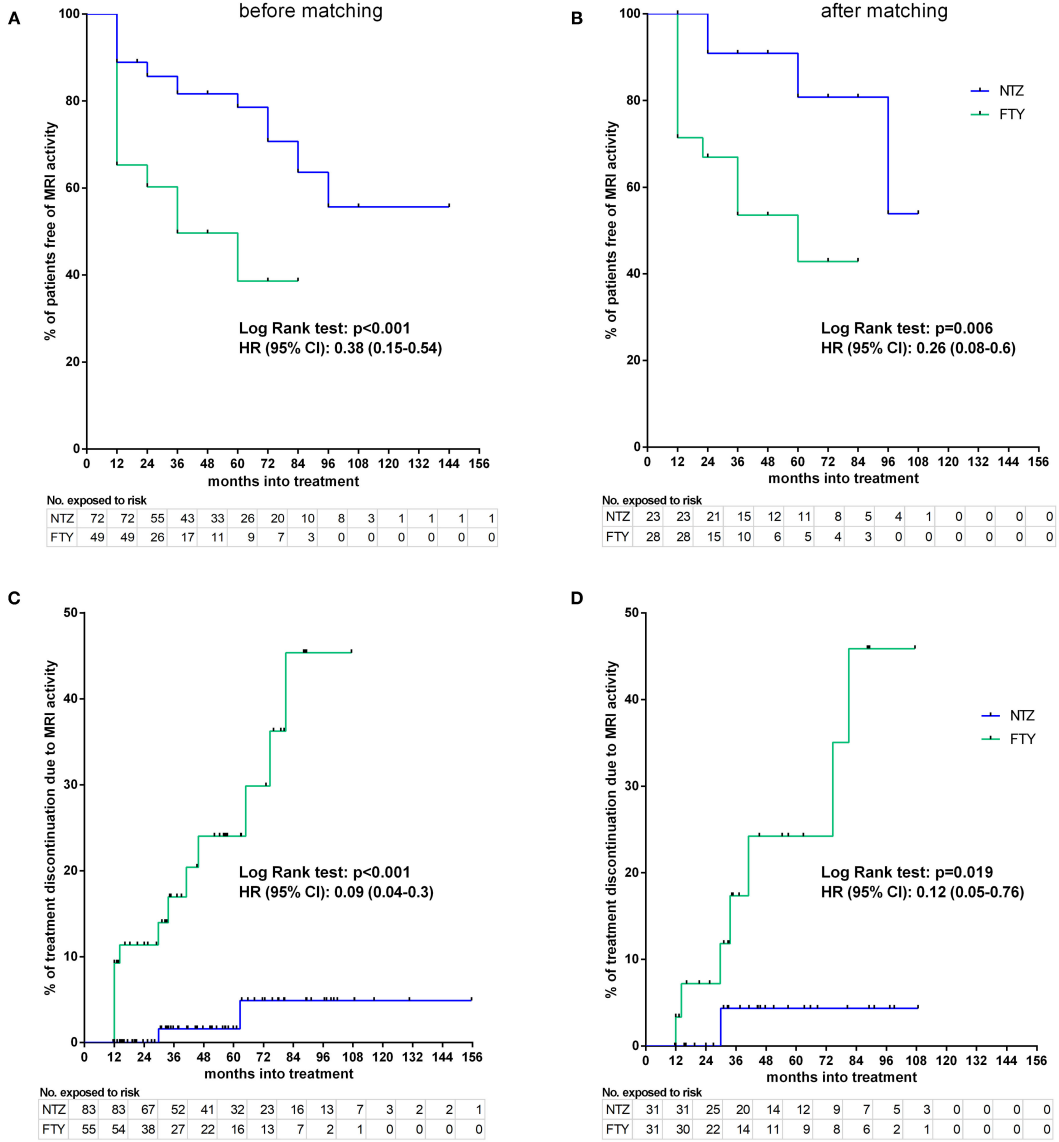
Kaplan–Meier survival estimates for the time to MRI activity **(A,B)** and time to treatment discontinuation due to MRI activity **(C,D)** before **(A,C)** and after **(B,D)** matching. HR, hazard ratio; CI, confidence interval; No., number; NTZ, natalizumab; FTY, fingolimod.

Before matching, NTZ was superior with respect to time to EDSS reduction (% of patients with disability improvement) (HR: 4.76, 95% CI: 1.23–8.67, Log Rank test *p* = 0.02) (Figure 6), time to relapse (% of patients free of relapse) (HR: 0.42, 95% CI: 0.18–0.86, Log Rank test *p* = 0.021) (Figure 7), time to MRI activity (% of patients free of MRI activity) (HR: 0.38, 95% CI: 0.15–0.54, Log Rank test *p* < 0.001), and time to treatment discontinuation due to MRI activity(HR: 0.09, 95% CI: 0.04–0.3, Log Rank test *p* < 0.001) (Figure 8), whereas a tendency toward NTZ superiority was shown for time to treatment discontinuation due to clinical activity (HR: 0.47, 95% CI: 0.17–1.13, Log Rank test *p* = 0.065) (Figure 7), without reaching statistical significance.

Sensitivity analyses were conducted by comparing the two unmatched groups (NTZ and FTY) following adjustment either for PS (first sensitivity analysis) or for all covariates that were used for PS calculation (second sensitivity analysis) and are shown in Table 2. Overall, sensitivity analyses were in agreement with the main analysis for all survival endpoints with few exceptions: following adjustment for covariates and for PS in the comparison between unmatched groups, NTZ was superior with respect to time to relapse (adjusted for covariates HR: 4.29, 95% CI: 1.76–10.47, *p* = 0.001; adjusted for PS HR: 4.08, 95% CI: 1.7–9.8, *p* = 0.002), time to MRI activity (adjusted for covariates HR: 3.47, 95% CI: 1.68–7.17, *p* = 0.001; adjusted for PS HR: 3.05, 95% CI: 1.5–6.21, *p* = 0.002). and time to treatment discontinuation due to MRI activity (adjusted for covariates HR: 14.38, 95% CI: 2.95–70.1, *p* = 0.001; adjusted for PS HR: 13.86, 95% CI: 2.87–67, *p* = 0.001) (as in the main analysis), whereas a similar tendency was shown for time to EDSS reduction following the only adjustment for covariates (HR: 0.22, 95% CI: 0.05–1.09, *p* = 0.064) and for time to treatment discontinuation due to clinical activity following adjustment for covariates and for PS, but the difference did not reach statistical significance (adjusted for covariates HR: 2.71, 95% CI: 0.96–7.65, *p* = 0.06; adjusted for PS HR: 2.54, 95% CI: 0.91–7.1, *p* = 0.075).

**Table 2:** Hazard ratios and relative 95% confidence intervals using proportional hazards model adjusted (a) by all covariates used for propensity score calculation and (b) by propensity score for unmatched cohorts and adjusted by all covariates used for propensity score calculation for matched cohorts.

**Outcome**	**Model**	**Unmatched**			**Matched**		
		**HR**	**CI 95%**	***p***	**HR**	**CI 95%**	***p***
Time to EDSS reduction	Adjusted for PS	0.34	0.07–1.63	0.179	–	–	–
	Adjusted for covariates	**0.22**	**0.05–1.09**	**0.064**	0.46	0.08–2.66	0.389
Time to EDSS increase	Adjusted for PS	1.29	0.41–4.09	0.666	–	–	–
	Adjusted for covariates	1.43	0.46–4.44	0.542	1.42	0.37–5.37	0.609
Time to relapse	Adjusted for PS	**4.08**	**1.7–9.8**	**0.002**	–	–	–
	Adjusted for covariates	**4.29**	**1.76–10.47**	**0.001**	**5.29**	**1.32–21.29**	**0.019**
Time to treatment discontinuation due to clinical activity	Adjusted for PS	**2.54**	**0.91–7.1**	**0.075**	–	–	–
	Adjusted for covariates	**2.71**	**0.96–7.65**	**0.060**	**8.78**	**0.84–92.02**	**0.070**
Time to MRI activity	Adjusted for PS	**3.05**	**1.5–6.21**	**0.002**	–	–	–
	Adjusted for covariates	**3.47**	**1.68–7.17**	**0.001**	**4.38**	**1.73–16.31**	**0.028**
Time to treatment discontinuation due to MRI activity	Adjusted for PS	**13.86**	**2.87–67**	**0.001**	–	–	–
	Adjusted for covariates	**14.38**	**2.95–70.1**	**0.001**	**8.48**	**0.94-76.98**	**0.057**

*HR, hazard ratio; CI, confidence interval; EDSS, Expanded Disability Status Scale; MRI, magnetic resonance imaging. Comparisons with a p value <0.01 are indicated in bold*.

In the weighted analysis for the unmatched cohorts, the overall test for proportional hazards showed NTZ superiority compared to FTY with respect to time to relapse (Wald F = 3.8, *p* = 0.002), time to discontinuation due to clinical activity (Wald F = 2.69, *p* = 0.017), and time to discontinuation due to MRI activity (Wald F = 3.86, *p* = 0.001), whereas the two treatments were comparable with respect to time to disability improvement (Wald F = 1.52, *p* = 0.175), time to disability worsening (Wald F = 0.71, *p* = 0.642) and time to MRI activity (Wald F = 1.66, *p* = 0.137).

### Matched Cohorts

#### Disability

Following PS matching, the mean change from baseline EDSS did not differ from the NTZ- and the FTY-treated patients in annual follow-up time points (Figure 3). Moreover, the mean EDSS did not differ between the two groups in annual follow-up time points. Time to disability improvement was not different between NTZ and FTY treated patients (Figure 6). In adjusted analysis, a tendency for the severity of the activity at baseline MRI (defined as the number of new/newly enlarged and Gd+ lesions on the brain and cervical MRIs) to predict disability improvement was observed (HR: 1.14, range: 0.98–1.34; *p* = 0.097), but the difference did not reach statistical significance. Time to disability worsening was not different between matched NTZ- and FTY-treated patients (Figure 6).

#### Relapse Activity

In the matched cohorts, both treatments resulted in profound mean ARR reduction at point estimates, compared to baseline (Figure 4). Moreover, with respect to the direct NTZ/FTY comparison, the mean ARR was significantly lower in the NTZ group compared to FTY at year 0–1 (NTZ vs. FTY, 0 vs. 0.13 ± 0.06, *p* = 0.04), at year 0–2 (NTZ vs. FTY, 0.04 ± 0.03 vs. 0.19 ± 0.05, *p* = 0.15) and with reference to the overall treatment duration (NTZ vs. FTY, 0.03 ± 0.02 vs. 0.13 ± 0.04, *p* = 0.03), whereas a tendency toward NTZ superiority was also evident at year 1–2 (NTZ vs. FTY, 0.08 ± 0.06 vs. 0.29 ± 0.1, *p* = 0.07) without reaching statistical significance (Figure 4). A significantly higher proportion of patients remained free of relapse in the NTZ group, compared to FTY (HR: 0.25, 95% CI: 0.08–0.8, Log Rank test *p* = 0.021) (Figure 7). With respect to treatment discontinuation due to clinical activity, a tendency toward NTZ superiority was evident, compared to FTY (HR: 0.23, 95% CI: 0.05–1.15, Log Rank test *p* = 0.073) (Figure 7), without reaching statistical significance. These differences were observed also following sensitivity analysis adjusted for covariates between the matched groups (Table 2).

#### MRI Activity

In the matched cohorts, both treatments resulted in a reduced mean number of new/enlarging T2 and Gd+ lesion reduction at point estimates compared to baseline in the brain (Figure 5) and cervical MRI (Supplementary Figure 1). Moreover, with respect to the direct NTZ/FTY comparison, the number of brain new/newly enlarged T2 lesions was lower for NTZ-treated patients at year 1 (NTZ vs. FTY 0 vs. 0.42 ± 0.19, *p* = 0.021) and the number of Gd+ lesions was lower for NTZ-treated patients at year 2 (NTZ vs. FTY 0 vs. 0.6 ± 0.39, *p* = 0.048), whereas a similar tendency was observed for NTZ-treated patients at year 1 (NTZ vs. FTY 0 vs. 0.09 ± 0.05, *p* = 0.078) (Figure 5), without reaching statistical significance. With respect to cervical MRI, the number of brain new/newly enlarged T2 lesions, and the number of Gd+ lesions did not differ between NTZ- and FTY-treated patients at annual follow-up time points (Supplementary Figure 1). Also, in the comparison between matched groups, the proportion of patients free of MRI activity was significantly higher for NTZ-treated patients compared to FTY (HR: 0.26, 95% CI: 0.08–0.6, Log Rank test *p* = 0.006) (Figure 8). Similarly, treatment discontinuation due to MRI activity was significantly higher for FTY-treated patients compared to NTZ (HR: 0.12, 95% CI: 0.05–0.76, Log Rank test *p* = 0.019) (Figure 8). In adjusted analysis, the results were similar to the main analysis with respect to time to MRI activity and the time of treatment discontinuation due to MRI activity (Table 2).

## Discussion

Early switch from first- to second-line DMTs in patients with highly active RRMS has been advocated as a strategy associated with favorable disease outcomes (30). Moreover, remaining free of relapse following the switch has been linked with improved persistence to the DMT (31), a factor also contributing to favorable overall disease prognosis. Natalizumab and FTY are highly effective DMTs in reducing relapse and radiological activity (1, 2). Although their use in RRMS is subjected to limitations due to safety issues, both treatments are considered to exhibit a more favorable safety profile compared to the newly available highly effective treatments indicated for highly-active RRMS and Relapsing Multiple Sclerosis (RMS), such as cladribine, alemtuzumab, and ocrelizumab, respectively (11, 13). In this respect, NTZ and FTY remain central in the management of highly active RRMS, and the availability of real-world, long-term safety and efficacy data is, therefore, crucial. The recent publication of 10-year real-world data regarding the safety and efficacy of natalizumab partly addresses this need. However, long-term comparative studies on the safety and efficacy of NTZ vs. FTY are expected to facilitate treatment decision upon switch from first- to second-line DMTs, especially when a newer highly effective treatment is not primarily considered, and to better characterize baseline patients' characteristics linked to optimal treatment response.

Due to the fact that direct comparative randomized prospective studies of NTZ vs. FTY are not available, treatment allocation is primarily based on empirical knowledge and real-world experience. Few post-marketing studies have retrospectively addressed issues with respect to NTZ vs. FTY comparative safety and efficacy, but the follow-up period is short at ~2 years (17–26) with the exception of one study with a total follow-up up to 4 years (27). However, also in this study, following PS matching, the mean follow-up time was ~1.8 years (27). In our study, the mean follow-up time for NTZ and for FTY was ~4.5 and 3.5 years, respectively, in unmatched and matched groups. The shorter follow-up period for FTY-treated patients is likely attributed to the earlier market availability of NTZ, as few patients in the NTZ-treated group had an especially long period of follow-up (15 patients: 7 years, 12 patients: 8 years, 6 patients: 9 years). Similarly, for the FTY-treated group, a long follow-up period was as follows: 15 patients: 5 years, 12 patients: 6 years, 7 patients: 7 years. The maximum follow-up period was 13 years for one patient under NTZ and 9 years for one patient under FTY.

Before PS matching, NTZ-treated patients exhibited higher mean ARR in the year before NTZ onset and higher mean EDSS score, compared to FTY-treated patients. This is in accordance with previous studies (17, 18, 20, 27). The higher mean ARR before treatment initiation may in fact indicate two factors that contribute toward NTZ or FTY treatment choice: (a) NTZ may be initiated preferentially, compared to FTY, in patients with more highly active disease due to the drug's documented capability toward rapid control of disease activity, compared to FTY [as indicated by a REVEAL study, in spite of its early discontinuation due to non-efficacy/non-safety issues (32, 33) and the recently published long-term follow-up safety and effectiveness study on NTZ (34)], and (b) FTY is more readily initiated to patients with relatively less highly active disease due to the more appealing route of administration and relatively low PML concern compared to NTZ. As previously proposed, higher mean EDSS at NTZ onset may indicate disability accumulation due to increased disease activity over the previous year. This observation is further confirmed by the analysis of unmatched groups in our study, according to which NTZ was superior to FTY with respect to disability improvement. In the analysis following PS matching, according to which baseline EDSS and ARR in the year before treatment onset did not differ between the two groups, NTZ was also superior, but the difference did not reach statistical significance. These results indicate that the superiority of NTZ with respect to disability improvement in the unmatched analysis is primarily attributed to patients with especially highly active disease and increased disability accumulation before NTZ onset, a group of patients for whom the sustained and/or reduced degree of disability is of special importance due to the higher burden over the quality of life.

In the matched analysis, NTZ was superior to FTY with respect to relapses (time to first relapse under treatment), as well as with respect to the time to MRI activity under treatment and treatment discontinuation due to MRI activity. Our results are in accordance with previous studies (17, 18, 20, 27) and are further supported by the sensitivity analyses performed in the unmatched and matched groups. The main reason for treatment discontinuation in the NTZ-treated group was PML concern, as in other studies. This fact, together with the lack of EMA guidelines for PML risk stratification in JCV seropositive NTZ-treated patients for treatment administration longer than 6 years renders post-marketing NTZ administration data with reference to longer follow-up especially rare. In our study, few JCV Ab seronegative patients insisted on continuing NTZ treatment following thorough information by the treating neurologist. These patients achieved long-standing clinical and radiological remission under NTZ. One seropositive patient developed PML shortly after NTZ discontinuation was suggested and PML was diagnosed at a pre-symptomatic phase on a routine MRI (35). In the FTY-treated group, treatment inefficacy and lymphopenia were the main reasons for treatment discontinuation. For both treatments, the time of discontinuation due to relapse and/or MRI activity was ~3 years. Also, in the FTY-treated group, patients with relatively long follow-up time achieved sustained remission of disease activity. These observations underline the need for longer post-marketing data on NTZ and FTY administration. More importantly, the need for NTZ-related PML stratification guidelines for longer follow-up appears of special importance, as evidence suggests that several patients may benefit from long-term NTZ administration.

Our study is subjected to limitations, such as its retrospective design, the lack of a central MRI facility, and the fact that it is a one-center study. However, the latter accounts for a more universal approach in treatment decisions and overall disease management. Moreover, although ARR in the year before NTZ/FTY onset, EDSS score at baseline (NTZ/FTY onset), and MRI measures of disease activity have been included as baseline characteristics, a treatment-naive status was not included as a binary variable in the baseline characteristics of the PS model. It should be noted, however, that the number of first-line DMTs has been included as a baseline characteristic in the PS model. In this respect, patients that did not receive first-line DMTs were represented as cases with a value of zero first-line DMTs prior to NTZ/FTY onset. Moreover, a profound reduction in the cohort sizes was evident following matching due to the fact that the two cohorts exhibited significant imbalance with respect to baseline characteristics, especially the ARR 1-year pre-NTZ/FTY treatment initiation and the EDSS. Following matching, the remaining cohorts were balanced, however, this improvement was at the expense of sample size. This is an inherent limitation of the real-world study setting. For reasons of transparency, we therefore present a comparison of unmatched and matched cohorts, with additional sensitivity and weighted analyses for the unmatched cohorts, as well as analysis of ARR, EDSS, and MRI parameters in a mean-change-from-baseline setting.

To conclude, our study provides real-world experience data on NTZ vs. FTY efficacy outcomes referencing a long follow-up period. Our results indicate NTZ superiority, compared to FTY, with respect to relapse and MRI activity outcomes, whereas the two treatments are comparable with respect to disability outcomes, in the analysis of the matched groups. These results are in accordance with previous studies. Moreover, the results of the present study also further support existing observations that NTZ evidently is empirically preferred for patients with more highly active RRMS with increased disability accumulation before treatment onset. It should be noted, however, that, in the frame of the present study, patients under NTZ were included who saw NTZ treatment initiation since 2007, as soon as NTZ became available, and who, due to the lack of alternative treatment plan, exhibited disability worsening before NTZ onset. The fact that these patients may achieve long-standing clinical and radiological remission upon prolonged treatment administration points toward the need for long follow-up data and universally accepted, evidence-based pharmacovigilance guidelines.

## Data Availability Statement

The original contributions presented in the study are included in the article/supplementary material, further inquiries can be directed to the corresponding author/s.

## Ethics Statement

The studies involving human participants were reviewed and approved by Bioethics' Committee of the School of Medicine of the Aristotle University of Thessaloniki (Approval Nr. 5321/23-2-2021). The patients/participants provided their written informed consent to participate in this study. The study was conducted in accordance to the Helsinki Declaration. All participants provided written informed consent.

## Author Contributions

MB performed the conception and design of the study, acquisition of data, analysis and interpretation, and critical revision of the manuscript for important intellectual content and gave final approval of the version to be submitted. CB, VG, S-AS, SK, TA, IN, PI, TK, IK, DP, and NG carried out the acquisition of data, analysis and interpretation, and critical revision of the manuscript for important intellectual content and gave final approval for the version to be submitted. All authors contributed to the article and approved the submitted version.

## Conflict of Interest

MB has received Travel support and/or research grants and/or lecture fees and/or advisory services from The Hellenic Foundation for Research and Innovation H.F.R.I., the Ministry of Education's Education and Lifelong Learning Program, the Hellenic Neurological Society, the Hellenic Academy of Neuroimmunology, Biogen Idec, Novartis, TEVA, Bayer, Genesis Pharma, Sanofi, Specifar, Roche, and Merck. CB has received Travel support and/or research grants and/or lecture fees and/or advisory services by Novartis, Bayer, Merck, Genesis, Sanofi, Specifar, Roche, Biogen, and Mylan. IN: conference fees and travel sponsorship: Bayer, specifar-TEVA, Novartis, Sanofi-Genzyme, Roche, and Mylan. Speaker honoraria: Merck, Sanofi-Genzyme, specifar-TEVA, Genesis Pharma, and Novartis. Honoraria for participation in advisory boards: Sanofi-Genzyme, Specifar-TEVA, Roche. TK: Honoraria and speaker's fees from Genesis-Biogen and Novartis. NG has received Travel support and/or research grants and/or lecture fees and/or advisory services: Novartis, Bayer, Merck, Genesis, Sanofi, Specifar, Roche, Biogen, TEVA, and Mylan. The remaining authors declare that the research was conducted in the absence of any commercial or financial relationships that could be construed as a potential conflict of interest.

## Publisher's Note

All claims expressed in this article are solely those of the authors and do not necessarily represent those of their affiliated organizations, or those of the publisher, the editors and the reviewers. Any product that may be evaluated in this article, or claim that may be made by its manufacturer, is not guaranteed or endorsed by the publisher.

## Supplementary Material

Supplementary Figure 1 Mean number of new/enlarging T2 lesions **(A,B)** and gadolinium-enhancing lesions **(C,D)** on annual cervical MRI of natalizumab and fingolimod cohorts at baseline and during the whole follow-up **(A,C)** before and **(B,D)** after matching. Statistical significance indicates mean change from baseline. NTZ, natalizumab; FTY, fingolimod; Nr, Number; Gd, gadolinium; ^*^*p* <0.05.

## Abbreviations

ARR: Annualized relapse rate
Ab: Antibody
DMTs: Disease Modifying Treatments
EMA: European Medicines Agency
EDSS: Expanded Disability Status Scale
FTY: Fingolimod
Gd+: Gadolinium
HRs: Hazard ratios
IFNs: Interferons
JCV: John Cunningham virus
MRI: Magnetic Resonance Imaging
MSD: Mean Standardized Difference
MS: Multiple sclerosis
NTZ: Natalizumab
PML: Progressive multifocal leukoencephalopathy
PS: Propensity score
RRMS: Relapsing–Remitting Multiple Sclerosis
SPMS: Secondary progressive multiple sclerosis.
