# Supplementary figures and images for: Long-Term Efficacy Outcomes of Natalizumab vs. Fingolimod in Patients With Highly Active Relapsing-Remitting Multiple Sclerosis: Real-World Data From a Multiple Sclerosis Reference Center
Source: Front Neurol. 2021 Aug 23;12:699844. doi: 10.3389/fneur.2021.699844 (PMC8419322; doi:10.3389/fneur.2021.699844)

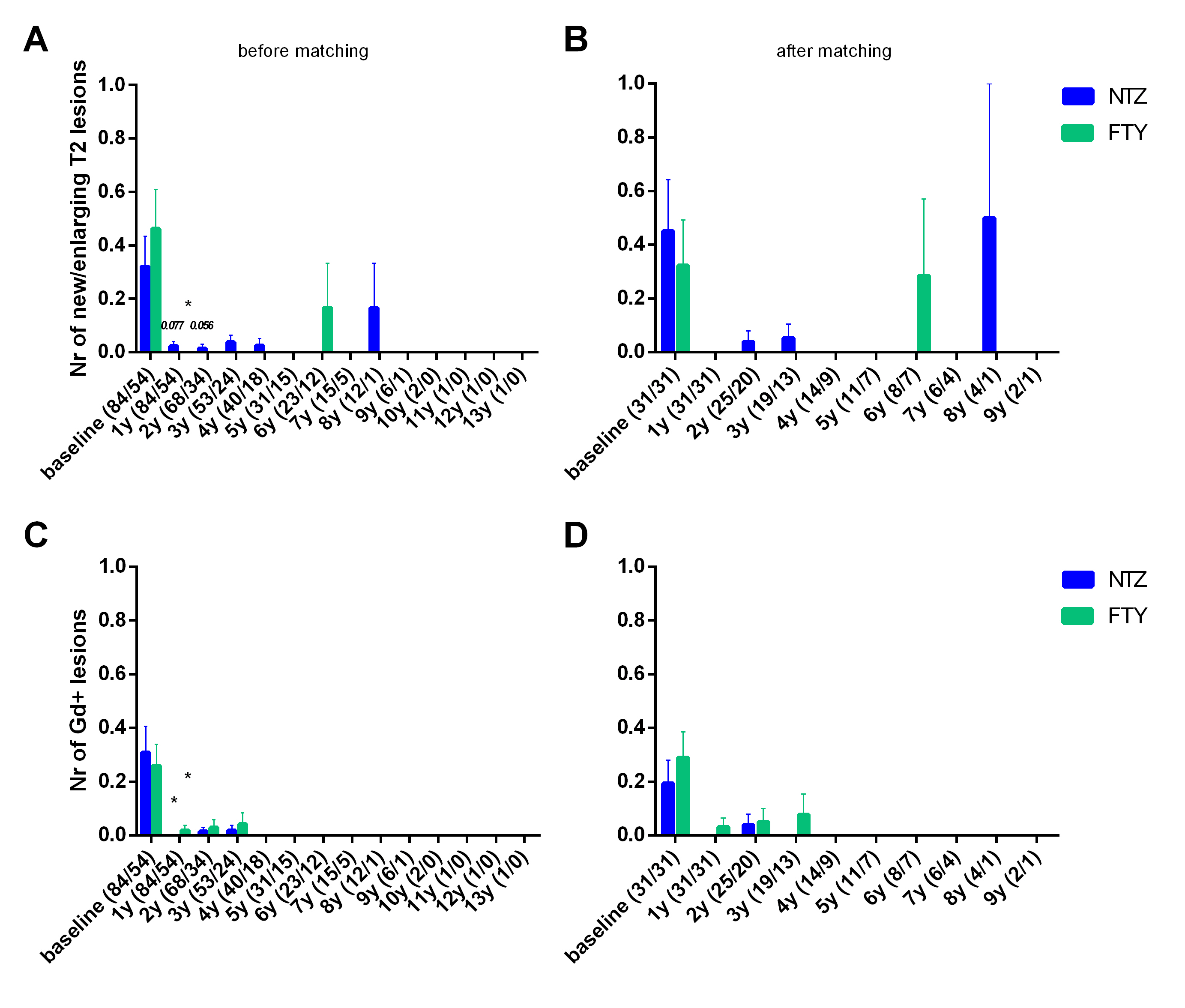

Supplement: Supplementary Figure 1 — Mean number of new/enlarging T2 lesions (A,B) and gadolinium-enhancing lesions (C,D) on annual cervical MRI of natalizumab and fingolimod cohorts at baseline and during the whole follow-up (A,C) before and (B,D) after matching. Statistical significance indicates mean change from baseline. NTZ, natalizumab; FTY, fingolimod; Nr, Number; Gd, gadolinium; *p <0.05. [file Image_1.JPEG]
